# Comparison of HEAR and HEART Scores for Major Adverse Cardiovascular Events

**DOI:** 10.7759/cureus.46721

**Published:** 2023-10-09

**Authors:** Umut Uyan

**Affiliations:** 1 Department of Cardiology, İzmir Ödemiş State Hospital, İzmir, TUR

**Keywords:** troponin, cardiovascular risk score, emergency department, chest pain, acute coronary syndrome

## Abstract

Background: Early identification of patients with low and high risk for acute coronary syndrome in the emergency department (ED) is important for process management and proper resource use. The aim of this study was to compare the HEAR and HEART scores to determine the risk for major adverse cardiovascular events (MACE) over 30 days.

Methods: Demographic data and clinical evaluations of the patients who presented to the ED with chest pain were recorded. ECGs were evaluated without knowing the clinical status of the patients. The HEART (including history, ECG, age, coronary risk factors, and troponin level) and HEAR (including four items with no troponin) risk scores were calculated. MACE was defined as all MI, all coronary revascularization procedures (PCI and CABG), all-cause death, cardiac arrest, cardiogenic shock, or life-threatening cardiac arrhythmias within 30 days. Patients with MACE were evaluated as Group 1, and patients without MACE were considered as Group 2, and the data from the two groups were compared.

Results: A total of 230 patients were included in the study. There were 56 (24.3%) patients with MACEs. According to the ROC analysis, the threshold value was determined as ≤3 for both scoring systems. According to this threshold value, sensitivity and specificity were found to be 0.77 and 0.78 for the HEAR score and 0.82 and 0.77 for the HEART score.

Conclusions: Although the HEAR and HEART scoring systems are useful for the management of patients with chest pain in the ED, the HEART score was evaluated to be more effective.

## Introduction

The goal for patients admitted to the ED with chest pain is to diagnose not only those at risk of acute coronary syndrome (ACS) but also those who are not in need of urgent care, are low-risk, or may not even have chest-pain-related cardiac issues [1]. ST-elevation myocardial infarction (STEMI) is easy to diagnose, but distinguishing ACS patients without ST elevation is more difficult. International guidelines [2] recommend that patients who are admitted to ES with chest pain should be evaluated using risk stratification tools or risk scoring systems. Since there is no precise definition or algorithm for patients with chest pain at risk of ACS, cardiac marker evaluation is performed for almost all patients. Objective risk scoring can help clinicians determine which patients need more inpatient testing and which patients can be safely discharged. The heart score is valuable in the risk stratification of patients presenting with chest pain. It is based on five elements, including history, electrocardiography, age, coronary risk factors, and troponin level. Patients with a risk score of three or less are considered low-risk, while a score greater than seven is considered high-risk, and those between these two values are regarded as having moderate risk for major adverse cardiac events (MACE). The HEAR score, the sum of four items with no troponin, can be used to lower phlebotomies for cardiac troponin T (cTnt) measurements while maintaining the HEART score safety profile. The aim of the present study is to compare the HEAR and HEART scoring systems for patients with chest pain and determine MACE for these patients for 30-day periods.

## Materials and methods

This study was planned with a prospective design to examine data from a provincial public hospital ED. This study was approved by the Katip Çelebi University Non-invasive Clinical Research Ethics Committee with the decision numbered 0318 on 16.06.2022, with the expectation of informed consent. Patients who applied to our ED due to chest pain diagnosed with acute coronary syndrome that was not related to trauma or cardiac arrest were included. The following information was recorded via a form prepared for patients admitted because of chest pain: demographic data and contact information; information related to the systemic diseases the patients suffered from, drugs they used, and their clinical histories; the types, durations and spreading patterns of their chest pain and accompanying symptoms; history of tobacco use; height and weight; troponin levels and vital signs; and clinical outcomes. The ECG results were evaluated by a cardiology specialist who had no prior information about their clinical status. The exclusion criteria were as follows: age < 18 years, having experienced an acute infection with fever (temperature > 38 C), an estimated glomerular filtration rate < 30 mL/min per 1.73 m2, known active inflammatory diseases, suspected myocarditis or pericarditis, and known severe liver disease. The primary endpoint was patients referred to the ED for STEMI and coronary revascularization at the time of their first admission to the ED. The secondary endpoint was repeated admission within 30 days of follow-up. Patients were contacted by phone, and information about whether they were admitted to another hospital, whether a cardiac intervention had been performed, or whether the patient had died was gathered. Patients who refused to be treated, who were admitted for inpatient care or to intensive care units for other reasons, who did not agree to participate, who had missing data in their patient files, or who could not be reached by phone after 30 days were excluded. Since our hospital information system is connected automatically to the state’s social security center, information about admission to other hospitals, performed procedures, and deceased patients is readily available. MACE was defined as all MI, all coronary revascularization procedures (PCI and CABG), all-cause death, cardiac arrest, cardiogenic shock, or life-threatening cardiac arrhythmias within 30 days. Patients with MACE were evaluated as Group 1, and patients without MACE were considered as Group 2. The HEAR and HEART risk scores were calculated. The heart evaluates the five main factors of history: ECG, age, risk factors, and troponin level. Each factor can receive up to 2 points. The HEAR evaluates the four main factors of history: ECG, age, and risk factors. HEART scores of 0-3, 4-6, and 7-10 were evaluated as reflecting low, medium, and high risk, respectively, while HEAR scores of 0-3, 4-6, and 7-8 were evaluated as low, medium, and high risk, respectively.

Statistical analysis

IBM Corp. Released 2013. IBM SPSS Statistics for Windows, Version 22.0. Armonk, NY: IBM Corp was utilized for statistical analysis. For the descriptive statistics, frequencies and percentages were given for categorical variables, and mean, standard deviation (SD) and median and range (minimum-maximum) values were given for numerical variables. The association between two categorical variables was analyzed with Pearson’s chi squared test. Group comparisons for numerical variables were performed with the independent sample t-test or Mann-Whitney U test. The receiver operating characteristic (ROC) curve was used to evaluate the scores’ ability to classify MACE status.

## Results

**Figure 1:**
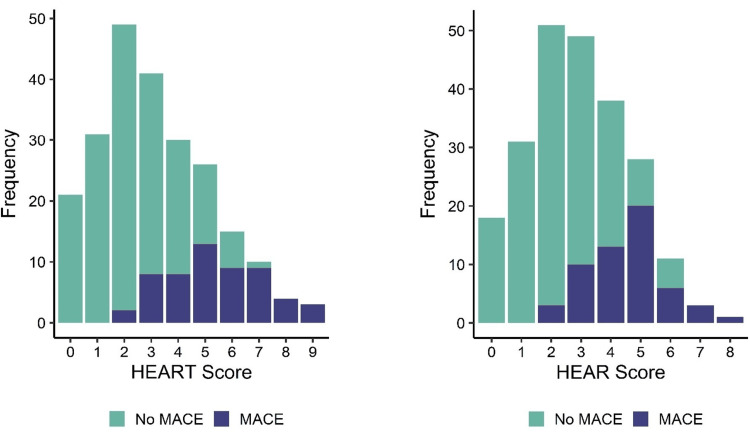
Incidences of HEAR-HEART score

**Figure 2:**
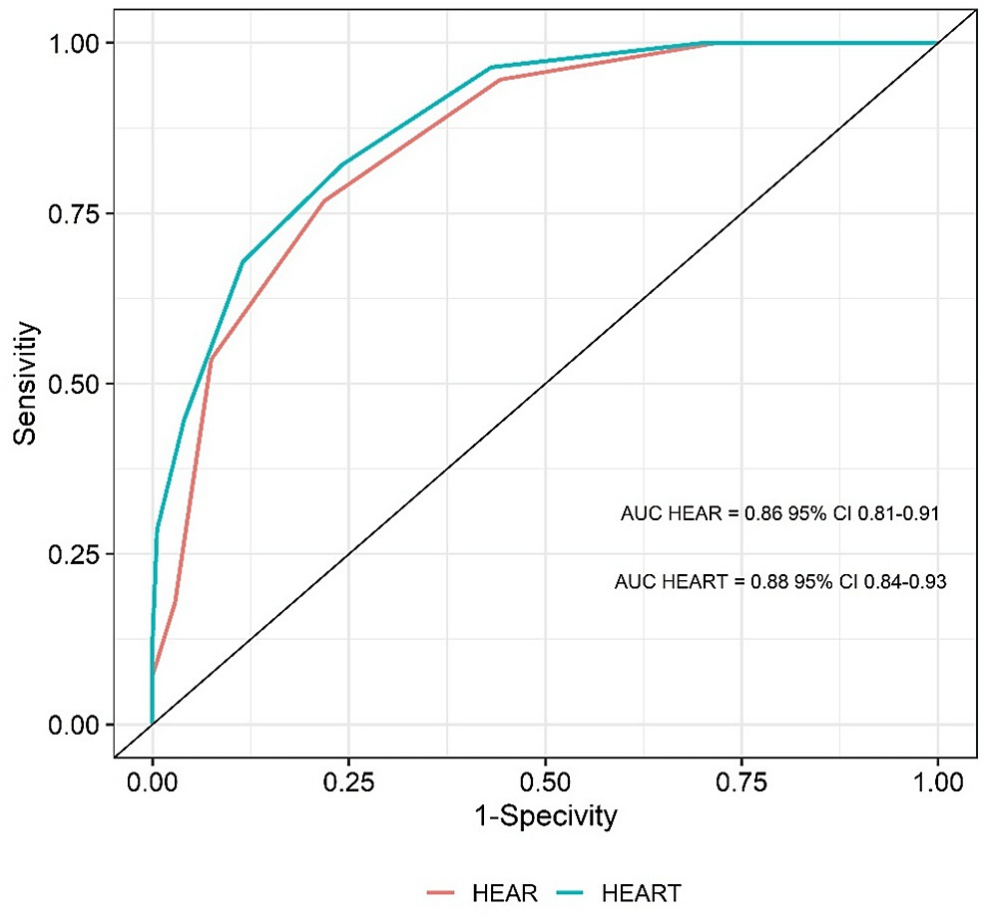
ROC analysis of the sensitivity and specificity ratios for the HEAR-HEART scores

Of the 255 patients who were admitted to the ED with chest pain unrelated to trauma or cardiac arrest, 25 patients who refused to be treated, were admitted to inpatient care or intensive care units, did not agree to participate in the study, or could not be contacted after 30 days were excluded. Statistical analyses were performed for the remaining 230 patients. The average age of these patients was 56.9 years, and 65.2% of the patients were male. There were 56 (24.3%) patients with MACEs (Group 1) and 174 (74.7%) patients without MACEs (Group 2). The distributions of age, sex, comorbid factors, and clinical histories of the groups with or without MACEs are presented in Table 1. While troponin levels, ECG results, and histories were statistically significantly different between groups, age and risk factors did not show statistically significant differences between these groups (Table 2). The average scores for the HEAR and HEART of the group that did no MACEs were found to be both 2.4. The average scores of Group 1 and Group 2 were statistically significantly different for the HEAR (4.5± 1.6 in Group 1) and HEART (5.4± 1.8 in Group 2) systems (p<0.001). Figure 1 presents the distributions of the HEAR and HEART scores. According to the ROC analysis, the heart was more effective than hearing. As shown in Figure 2, the AUC for HEAR was found to be 0.86, with a 95% CI of 0.81-0.91, while that for HEART was 0.88, with a 95% CI of 0.84-0.93, respectively. Sensitivity and specificity values calculated for that threshold were 0.77 and 0.78, respectively, for the ear and 0.82 and 0.77, respectively, for the heart. MACE rates for patients scored as being of low, medium, and high risk were 8%, 50%, and 100%, respectively, by HEAR, and 7%, 42%, and 94%, respectively, by HEART. There was a statistically significant difference (p<0.001) between the scoring systems in terms of the distribution of MACEs according to risk groups (Table 3).

**Table 1:** Baseline characteristics of the groups MACE: Major adverse cardiovascular events

	No MACE	MACE	Total	p-value
Age (years)	55.7± 16.9	60.5±12.6	59.6 ± 16.1	0.027
Gender Male, n (%)	108 (62.1%)	42 (75%)	150 (65.2%)	0.077
Diabetes Mellitus, n (%)	37 (21.3%)	17 (30.4%)	54 (23.5%)	0.163
Smoking, n (%)	61(34.1%)	17 (30.4%)	78 (33.9%)	0.518
Hyperlipidemia, n (%)	30 (17.2%)	16 (28.6%)	46 (20.0%)	0.065
Hypertension, n (%)	67 (38.5%)	25 (44.6%)	92 (40.0%)	0.415
Family history, n (%)	31 (17.8%)	16 (28.6%)	47 (20.4%)	0.083
Obesity, n (%)	37 (21.3%)	21 (37.5%)	58 (25.2%)	0.015
History of myocardial infarction, n (%)	34 (19.5%)	9 (16.1%)	43 (18.7%)	0.563
History of angiography	45 (25.9%)	18 (32.1%)	63 (27.4%)	0.359
History of coronary artery bypass graft, n (%)	13 (7.5%)	5 (8.9%)	18 (7.8%)	0.724
History of cerebral vascular disease, n (%)	5 (2.9%)	1 (1.8%)	6 (2.6%)	0.657
Peripheral vascular disease, n (%)	19 (10.9%)	3 (5.4%)	22 (9.6%)	0.218

**Table 2:** Evaluation of score factors MACE: Major adverse cardiovascular events

	No MACE n (%)	MACE n (%)	Total n (%)	p-value
Troponin score				
0	162 (93.1)	30 (53.6)	192 (83.5)	<0.001
1	9 (5.2)	6 (10.7)	15 (6.5)	
2	3 (1.7)	20 (35.7)	23 (10.0)	
ECG score				
0	133 (76.4)	24 (42.9)	157 (68.3)	<0.001
1	20 (11.5)	12 (21.4)	32 (13.9)	
2	21 (12.1)	20 (35.7)	41 (17.8)	
Age score				
<45	51 (29.3)	5 (8.9)	56 (24.3)	0.006
45-65	67 (38.5)	31 (55.4)	98 (42.6)	
>65	56 (32.2)	20 (35.7)	76 (33.0)	
History score				
0	143 (82.2)	3 (5.4)	146 (63.5)	<0.001
1	29 (16.7)	22 (39.3)	31 (55.4)	
2	2 (1.1)	31 (55.4)	33 (14.3)	
Risk factors score				
0	62 (35.6)	20 (35.7)	82 (35.7)	0.805
1	84 (48.3)	25 (44.6)	109 (47.4)	
2	28 (16.1)	11 (19.6)	39 (17.0)	

**Table 3:** Risk stratification of groups MACE: Major adverse cardiovascular events

	No MACE (n:156) n (%)	MACE (n:74) n (%)	Total (n:230) n (%)	Incidence Rate of MACE (%)	p- value
HEART					
Low	132 (75.9)	10 (17.9)	142 (61.7)	7	<0.001
Intermediate	41 (23.6)	36 (53.6)	71 (30.9)	42	
High	1 (0.6)	16 (28.6)	17 (7.4)	94	
HEAR					
Low	136 (78.2)	13 (23.2)	149 (64.8)	8	<0.001
Intermediate	38 (21.8)	39 (69.6)	77 (33.5)	50	
High	0 (0.0)	4 (7.1)	4 (1.7)	100	

## Discussion

This study aimed to compare the HEAR and HEART for patients admitted to ES with chest pain and evaluate the risk of MACEs for a 30-day period. The 2014 AHA/ACC guidelines recommend that serial troponin measurements be taken at admission and three to six hours after the onset of symptoms in all patients for diagnosis in ACS management [3]. However, there is no universally accepted and applied risk scoring system in ED. This research fits within the existing literature by adding evidence to the utility of risk scoring systems, specifically HEAR and HEART, in the context of patients presenting with chest pain. Our study is consistent with previous studies investigating risk assessment tools to identify patients at risk for MACE. While the findings support the use of these scoring systems, they also contribute to the ongoing debate about the most effective methods for risk stratification in emergency department settings. The study concludes that both the HEAR and HEART scoring systems are reliable for use in managing patients with chest pain in the emergency department. However, the HEART score is found to be slightly more effective. 

Six et al. found that, of 122 patients suffering from chest pain, those who scored 0-3 points could be immediately discharged with a risk rate of 2.5%, those who scored 4-6 points warranted clinical follow-up with a risk rate of 20.3%, and those who scored ≥7 points required early invasive intervention with a risk rate of 72.7% according to the endpoint (acute myocardial infarction (AMI), percutaneous coronary intervention, coronary artery bypass grafting, and death) [3]. In our study, these values were found to be 7%, 42% and 94%, respectively. The study, which determined that the mean score of patients who reached the relevant endpoints was 6.51 while that of those who did not was 3.71, noted that patients were followed for a period of about three months [3]. In our study, the mean score of the group that developed MACEs was found to be 2.4, while that of those who did not was found to be 5.4. As risk score values increased, the risk of developing MACEs also increased. The difference between the findings of our study and those of Six et al. may be due to the difference in follow-up periods. O’Rielly et al. stated that 202 (17.6%) patients with HEAR scores of ≤1 had a very low risk of MACEs in a 30-day period with a sensitivity of 99.2%. They evaluated the risk scoring and endpoints of the patients after 30 days according to the presence of coronary artery disease and reported that approximately 58% of patients belonged to the subgroup with a HEAR score of ≤3. In a different study, the sensitivity of MACEs was found to be 99.2% in the 30-day follow-up of patients [4]. In our study, 78.2% of patients who did not develop MACEs were found to be in the low-risk group, and our study found the sensitivity for MACEs to be 77%. We believe this difference may stem from the fact that we defined the low-risk group as having a score of ≤3. 

Backus et al. [5] determined the average HEART score of a total of 2388 patients to be 4.4; that of the group that developed MACEs was 6.54; and that of the group that did not was 3.96. In our study, the heart score for the group that developed MACEs was found to be 5.4, while that for the group that did not was found to be 2.4. The mean HEAR and HEART scores were 2.9 and 3.1, respectively. That study included 407 patients with MACEs and 1981 patients without MACEs [5]. They found the MACE development rate to be 1.7%, 16.6%, and 50.1 for low (0-3), medium (4-6), and high (7-9) HEART scores, respectively [5]. In our study, while 61.7%, 30.9%, and 17% of the total patient population were scored as being of low, medium, and high risk, respectively, they developed MACEs at rates of 7%, 42% and 94%, respectively. Our study population is smaller than that of Backus et al., in which the patients were followed for six weeks, but the mean scores in the groups with and without MACEs are comparable between these studies. The scores were higher in the group that developed MACEs, similar to the results of our study. In general, when we compare our results with those of Backus et al. [5], the rate of MACEs is significantly higher for the high-risk group. Moumneh et al. [6] evaluated the reliability of the CARE rule and HEART scoring system in their prospective study, in which they determined the MACE rate of 641 patients with chest pain in a six-week period to be 9.5%. In their study, the HEART score for the group that developed MACEs was found to be seven, while that for the group that did not was 2.9, and the mean score of the total population was determined to be 3.4. The CARE scores of the same groups were found to be 5.7, 2.6, and 2.9, respectively. In our study, these values were determined to be 4.5, 2.4, and 2.9, respectively. The area under the ROC curve was 0.91 for the CARE criteria and 0.95 for HEART scores [6]. In our study, these values were 0.86 and 0.88, respectively. These values suggest that the scoring systems are reliable. Moumneh et al. stated that the sensitivity levels for CARE values of <2 and HEART values of <4, considered as the thresholds for low-risk groups, were 100% [6]. Sensitivity levels in our study were over 75%. This difference may be due to the low-risk group being considered as the group receiving a score of 3 or lower. Rad et al. showed through the evaluation of data related to HEAR scores that the sensitivity levels for HEAR cut-offs of <2, <3, and <4 in ES were 99.03%, 97.54%, and 91.80%, respectively. The net present value (NPVs) for the above thresholds were 99.84%, 99.75%, and 99.57%, respectively. The negative probability ratio for HEAR scores of <2 was 0.07. They showed that a HEAR score of <2 is appropriate for an early discharge strategy in ED [7]. In the present study, the threshold value was found to be ≤3. Furthermore, the NPV value according to HEAR scores was 0.91 and for HEART scores was 0.93. These values indicate that these scoring systems are reliable for use among patients in the low-risk group.

Moumneh et al. [8] stated that 1.1% of the patients had STEMI or died, three of 4106 (0,07%) patients with HEAR scores of <2 died, and two had AMI, all during the 30-day follow-up period. Sensitivity and specificity were found to be 97.9% and 18.8%, respectively. The results suggest that low HEAR scores can be used to accurately identify patients with a very low 30-day risk of AMI or death and may be used to identify a group of patients for whom troponin testing can be excluded [8]. The fact that the sensitivity was determined at 77% in our study may be due to the different risk scoring system we employed. Smith et al. [9] concluded that patients with HEAR scores of 0-1 represent a very low-risk group who may not require troponin tests to obtain a missed MACE rate of <1%. They found that MACEs occurred in 0.9% of patients with HEAR scores of ≤1 and a sensitivity of 97.8% during the 30-day follow-up period. This finding suggests that troponin tests are of low benefit for this population. In our study, we found sensitivity to be 77% for patients with HEAR scores of ≤3, and our MACE rate was found to be 8% in this group. This difference may be since risk groups were categorized differently. Otsuka et al. [10] found that the incidence rates of MACEs among patients of low, medium, and high risk were 0%, 23.2%, and 63.6%, respectively, by heart analysis and 4.7%, 22.9%, and 62.5%, respectively, by hearing analysis. In this study, NPVs for MACEs were found to be high for both systems. These values indicate that both scoring systems are effective in determining low-risk patients with chest pain in terms of MACE incidence. Sharp et al. assigned ≤5 points as the threshold value for patients with a low risk of adverse events for 30 days. Mortality, or AMI, at the end of the 30-day follow-up period was assessed as a risk. In the group of patients with heart scores of ≤5, the risk was less than 1%. The authors stated that setting a HEART score of ≤5 as the low-risk threshold is a rational approach that can prevent unnecessary testing and revascularization procedures in the hospital [11-15]. In our study, we set this threshold value at three. The difference between the mentioned study and our study may be due to mortality, AMI, and revascularization procedures being considered as risks. Patients in the low-risk group seem to be very unlikely to die or develop AMI.

Limitations

Although our study was prospective, the accuracy of the answers given by the participants at the time of admission and at the end of 30 days is the limitation of this study. The number of patients was low. Accessing information via the hospital automation system was another limitation, in that it limits control in cases where the data inputs of this system are incorrect. Another limitation is that it was a single-center study, and coronary angiography results were not included. Angiography results could change the endpoint.

## Conclusions

Data in the literature and in our study indicate that the HEAR and HEART are reliable for use in the management of patients with chest pain. However, only low-risk groups have been considered in studies conducted on these scoring systems. Referral of high-risk patients from the ED to appropriate clinics for rapid further examination may be more useful to reduce the patient load.
